# Human mesenchymal stromal cell-secreted lactate induces M2-macrophage differentiation by metabolic reprogramming

**DOI:** 10.18632/oncotarget.8623

**Published:** 2016-04-06

**Authors:** Silvia Selleri, Panojot Bifsha, Sara Civini, Consiglia Pacelli, Mame Massar Dieng, William Lemieux, Ping Jin, Renée Bazin, Natacha Patey, Francesco M. Marincola, Florina Moldovan, Charlotte Zaouter, Louis-Eric Trudeau, Basma Benabdhalla, Isabelle Louis, Christian Beauséjour, David Stroncek, Françoise Le Deist, Elie Haddad

**Affiliations:** ^1^ CHU Sainte-Justine Research Center, Montreal, QC, Canada; ^2^ Department of Microbiology, Infectiology and Immunology, University of Montreal, Montreal, QC, Canada; ^3^ Department of Transfusion Medicine, Clinical Center, NIH, Bethesda, MD, USA; ^4^ Department of Pharmacology, University of Montreal, Montreal, QC, Canada; ^5^ Department of Biology, New York University, Abu Dhabi, United Arab Emirates; ^6^ Department of Research and Development, Héma-Québec, Québec, QC, Canada; ^7^ Department of Pathology, University of Montreal, Montreal, QC, Canada; ^8^ Sidra Medical and Research Center, Doha, Qatar; ^9^ Faculty of Dentistry, University of Montreal, Montreal, QC, Canada; ^10^ Department of Pediatrics, University of Montreal, Montreal, QC, Canada

**Keywords:** mesenchymal stromal cells, dendritic cell differentiation, M2-macrophages, lactate, metabolism, Immunology and Microbiology Section, Immune response, Immunity

## Abstract

Human mesenchymal stromal cells (MSC) have been shown to dampen immune response and promote tissue repair, but the underlying mechanisms are still under investigation. Herein, we demonstrate that umbilical cord-derived MSC (UC-MSC) alter the phenotype and function of monocyte-derived dendritic cells (DC) through lactate-mediated metabolic reprogramming. UC-MSC can secrete large quantities of lactate and, when present during monocyte-to-DC differentiation, induce instead the acquisition of M2-macrophage features in terms of morphology, surface markers, migratory properties and antigen presentation capacity. Microarray expression profiling indicates that UC-MSC modify the expression of metabolic-related genes and induce a M2-macrophage expression signature. Importantly, monocyte-derived DC obtained in presence of UC-MSC, polarize naïve allogeneic CD4^+^ T-cells into Th2 cells. Treatment of UC-MSC with an inhibitor of lactate dehydrogenase strongly decreases lactate concentration in culture supernatant and abrogates the effect on monocyte-to-DC differentiation. Metabolic analysis further revealed that UC-MSC decrease oxidative phosphorylation in differentiating monocytes while strongly increasing the spare respiratory capacity proportional to the amount of secreted lactate. Because both MSC and monocytes are recruited *in vivo* at the site of tissue damage and inflammation, we propose the local increase of lactate concentration induced by UC-MSC and the consequent enrichment in M2-macrophage generation as a mechanism to achieve immunomodulation.

## INTRODUCTION

Based on their immunomodulatory properties [1, 2], mesenchymal stromal cells (MSC) have been explored for the treatment of several immune-related diseases [3] such as graft *versus* host disease (GvHD) [4], Crohn's disease [5], multiple sclerosis [6] type-2 diabetes [7], and have been shown to induce tissue remodeling and repair [8–11]. Notwithstanding encouraging *in vitro* and *in vivo* results, the mechanisms underlying the MSC-mediated biological effects are still undefined [12–16]. Improving our knowledge on the mechanisms of action of MSC is an urgent need, especially because recent clinical trials using MSC infusion for the treatment of steroid resistant GvHD have provided positive results regarding overall response and survival [4, 17–19].

To investigate how MSC modulate the immune response, we focused on their effects on the differentiation and maturation of monocyte-derived dendritic cells (DC). DC are professional antigen presenting cells that play a key role in the activation and regulation of adoptive immune responses in both physiological and pathological conditions [20–23]. Upon uptake of antigens released by tissues (i.e. upon conditioning-induced damages in the case of GvHD), immature DC undergo maturation and migrate to draining lymph nodes where they present antigens and provide co-stimulation to donor's naïve T-cells by cell-cell interactions and the secretion of soluble factors (i.e. IL-12). As a consequence, activated T-cells acquire a Th1, IFNγ producing phenotype, proliferate and migrate to the inflamed damaged tissue where they elicit effector functions. Because DC are initiators of the immune cascade, targeting their activity is an appealing tool for the regulation of immune responses [20]. The redirection and tuning of myeloid cell differentiation is likely to be a natural way of adapting the immune response to local stimuli [24–26].

Here, we extensively investigated the changes induced by umbilical-cord (UC)-MSC on the differentiation and function of monocyte-derived DC. Based on our findings, we propose that UC-MSC divert the differentiation of monocyte-derived DC into M2-macrophages by metabolic reprogramming via lactate secretion. Due to the increasing interest in immune cell metabolism in both physiological and pathological conditions, and to the importance of DC and M2-macrophages in immune regulation, the possibility of manipulating myeloid cell plasticity by metabolic reprogramming represents an important step towards new clinically relevant therapies.

## RESULTS

### UC-MSC prevent the differentiation of GM-CSF/IL-4-treated monocytes into DC

*In vitro* stimulation of monocytes with GM-CSF and IL-4 promotes their differentiation into immature DC (iDC), and further stimulation with LPS induces DC maturation (mDC) [27, 28]. We investigated whether the presence of UC-MSC during the differentiation and maturation phase could alter the general properties of the resulting DC (here indicated as iDC^MSC^ and mDC^MSC^ respectively as shown in Figure S1).

**Figure 1:**
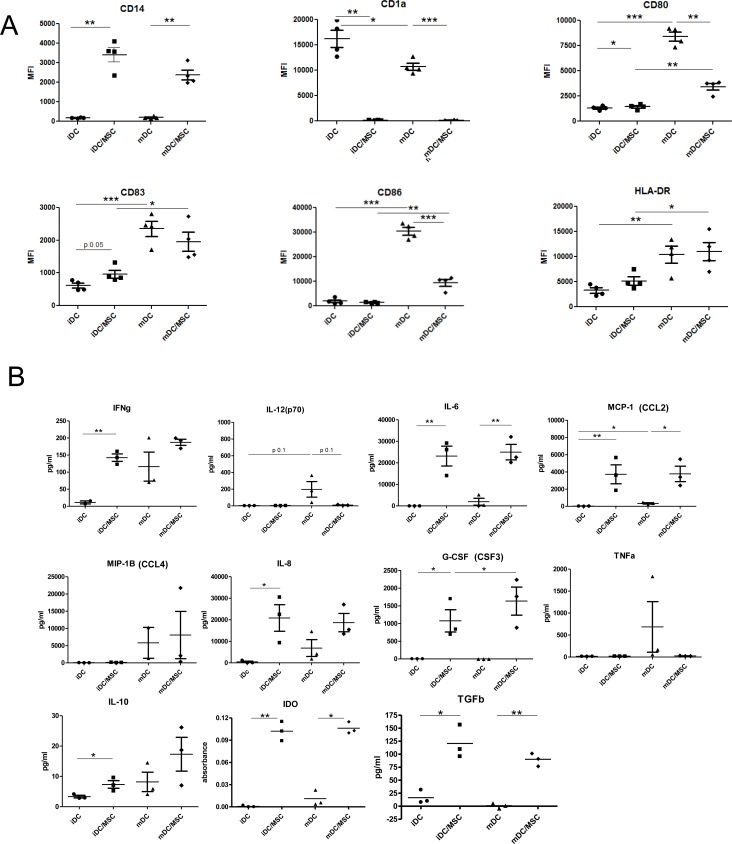
DC surface marker expression and cytokine secretion analysis from cultures of DC in presence or absence of UC-MSC **A.** Monocytes were differentiated in the presence or absence of primary UC-MSC. At the end of the culture, cells were harvested and surface markers were analyzed by flow cytometry. The expression of CD1a (DC marker), CD80 and CD86 (co-stimulatory molecules), HLA-DR (important for antigen presentation) and CD83 (maturation marker) were assessed, gating on 7AAD neg (live) cells. UC-MSC were excluded from the analysis based on their higher size and granularity in respect to DC. Statistics: unpaired t-test, *p < 0.05, **p < 0.01, ***p < 0.0001. iDC were compared with iDC^MSC^ and mDC, iDC^MSC^ with mDC^MSC^, mDC with mDC^MSC^. MFI: mean fluorescent intensity. *N* = 4 different primary UC-MSC. B) The supernatant from DC differentiated from monocytes and matured by LPS in the presence or absence of UC-MSC was analyzed for cytokine release. Data refers to 3 independent experiments, run in duplicate, using primary UC-MSC. For some of the samples only 2 experiments were considered valid by the analysis software. Statistics: unpaired t-test, 2 tails, *p < 0.05, **p < 0.01, ***p < 0.0001.

Flow cytometry analysis revealed that iDC were CD14^−^, CD1a^high^, CD80^low^, CD83^low^, CD86^low^, HLA-DR^+^, while mDC were CD14^−^, CD1a^high^, CD80^high^, CD83^+^, CD86^high^, HLA-DR^high^ (Figure 1A and Figure S2, S3). The presence of primary UC-MSC during DC differentiation deeply modified the cellular surface marker expression pattern. Indeed, in contrast to iDC, the phenotype of iDC^MSC^ was CD14^+^ and CD1a^−^ (Figure 1A) suggesting a profound modification of the nature of the obtained cells. In comparison to mDC, mDC^MSC^ showed a significantly lower expression of CD80 and CD86, and expressed CD14. MSC were excluded from the analysis based on their size and granularity. Of note, UC-MSC mediated their effect during the DC differentiation phase, as iDC^MSC^ were not able to acquire a mature phenotype even if UC-MSC were removed during the LPS-induced maturation phase, and iDC matured correctly in presence of UC-MSC during the maturation phase only (data not shown).

Significantly higher concentrations of IFNγ, IL-6, MCP-1, IL-8, G-CSF, TGFβ and IL-10 were detected in iDC^MSC^ *versus* iDC supernatants (Figure 1B). Compared to mDC, mDC^MSC^ supernatants were significantly enriched in IL-6, MCP-1, G-CSF and TGFβ, while IFNγ, IL-8 and IL-10 were increased albeit not significantly. The production of IL12 (p70), a hallmark of mature DC, was absent from the mDCMSC condition (Figure 1B) suggesting a profound alteration of cell identity induced by the presence of UC-MSC. In comparison to i/mDC, i/mDC^MSC^ supernatants contained a significantly higher concentration of the enzyme indoleamine 2,3-dioxygenase (IDO), which is typically secreted by MSC upon stimulation (Figure 1B). In all tested conditions, the levels of IL-5, IL-13, IL-17, IL-1B, IL-2 and IL-7 were below the detection threshold of 20 pg/ml (data not shown). Under these experimental settings, we cannot determine whether the high levels of IDO, IFNγ, IL-6, IL-8, MCP-1 and G-CSF secretion in the i/mDC^MSC^ conditions could be attributed to the i/mDC^MSC^, the MSC or a combination of both. Mirroring the surface marker expression analysis, these results show that i/mDC^MSC^ display a distinct cytokine secretion profile from i/mDC further confirming our observations that UC-MSC profoundly affect the canonical monocyte-to-DC differentiation process.

**Figure 2:**
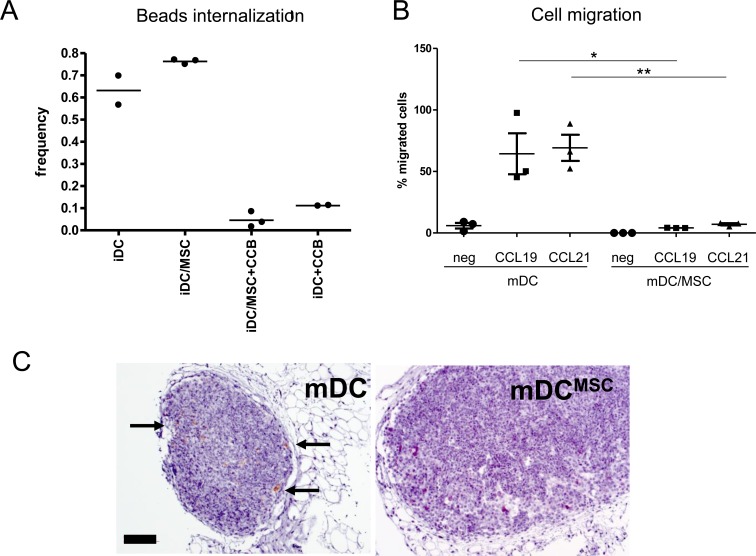
UC-MSC alter the function of monocyte-derived DC **A.** Zymosan-coated PhRodo beads up-take by immature DC obtained on the presence or absence of primary UC-MSC (iDC and iDC^MSC^). Three different donors were tested. CCB = cytochalasin-B (up-take inhibitor). **B.** mDC and mDC^MSC^ were harvested, counted and tested for their migratory capacity towards potent DC-attractant chemokines. The percentage of cells migrating *in vitro* towards medium only (circles) MIP3b = CCL19 (300 ng/ml, squares) and Exodus2 = CCL21 (500 ng/ml, triangles) is reported. MSC were eliminated from the culture before testing cellular migration. Statistics: unpaired t-test, 2 tails, *p < 0.05, **p < 0.01. Data were obtained from 3 independent experiments using 3 primary UC-MSC. **C.** Immunohistochemistry analysis of poplitear lymph node sections of mice upon footpad injection of either mDC or mDC^MSC^ (upon MSC depletion). The staining shows human CD45^+^ cells. The data were obtained 24 hours after the injection of 2×10^6^ cells. For these experiments the MSC cell line was used.

Antigen uptake and migratory capacity are typical characteristics of iDC and mDC, respectively. We observed that iDC^MSC^ cells were able to engulf Zymosan-coated beads with an efficiency comparable to that of iDC (Figure 2A), which indicates that iDC^MSC^ are functional antigen presenting cells. Moreover, we tested the *in vitro* capacity of LPS-matured cells to move towards chemoattractants in a transwell system. CCL19 and CCL21 are potent mDC attractants and generate the gradient necessary for mDC homing to the lymph nodes [29]. We observed that 55-60% of plated mDC were able to reach the attractants whereas mDC^MSC^ were unable to migrate (Figure 2B). To further support these findings *in vivo*, we took advantage of a mouse model already described for testing the potential of human DC migration [30]. We injected cells matured with LPS (mDC and mDC^MSC^) in the footpad of C57/B17 SCID mice and found that only mDC were able to reach the draining lymph node (Figure 2C).

### i/mDC^MSC^ have a M2-macrophage gene expression signature and morphology

**Figure 3:**
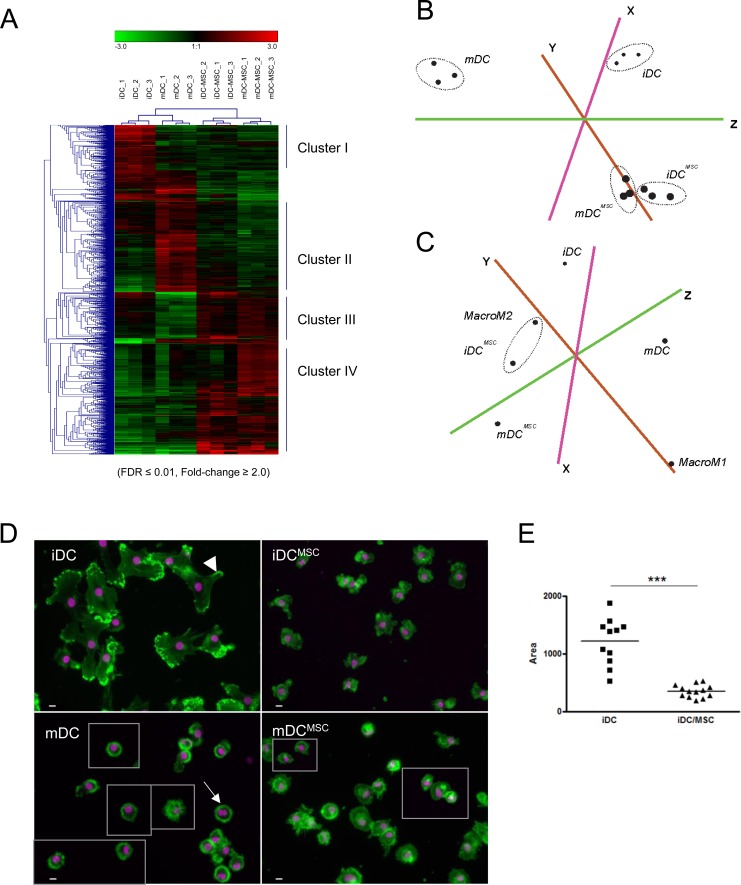
UC-MSC induce, in monocytes differentiating into DC, M2-macrophage expression signature and morphology Monocytes were differentiated and eventually matured in presence or absence of UC-MSC. Gene expression profile was investigated after MSC removal form the culture. **A.** Total gene lists with Loess-normalized array signals were pruned according to criteria of enrichment (Fold-change ≥2.0, FDR≤0.01, Array signal>60) between iDC/iDC^MSC^ or mDC/mDC^MSC^. Unique gene identifiers along with their normalized array signal values across samples (4 differentiation conditions, 3 donors per condition) were then submitted to Genesis tool for unbiased hierarchical clustering (average linkage option). Heat maps within each sample (column) indicate the relative expression value of a particular gene probe (row) compared to the averaged expression value of all the probes. Shades of red represent a higher signal compared to the average signal, whereas green shades represent a lower signal. **B.** Principal component analysis (PCA) of gene lists analyzed in panel A shows that iDC^MSC^ and mDC^MSC^ cluster very close together, while iDC and mDC are found farther from them and, as expected, apart from each other. **C.** PCA of our expression profiling data with publically-available data (GSE5099) indicates a strong similarity between iDC^MSC^ and monocyte-derived M2 macrophages. **D.** Fluorescence microscopy shows that the presence of UC-MSC during differentiation/maturation phase alters DC morphology (iDC^MSC^ and mDC^MSC^). For convenience, some cells were taken from distant part of the original picture and grouped; in this case, a light grey line indicates the border of the cropped field. In the upper left panel the arrowhead point to one of the cell protrusions typical of iDC and missing in iDC^MSC^. In the lower left panel the arrow points to the characteristic dendrites of mDC, missing in mDC^MSC^. Green: actin; pink: nuclei. Magnification 200x, bar: 10 microns. **E.** Area quantification analysis by ImageJ software shows a significant difference in size between iDC and iDC^MSC^. Statistics: unpaired t-test, ***p < 0.0001. Data obtained by using primary UC-MSC. Similar results were obtained when using an immortalized UC-MSC cell line (data not shown).

To extensively characterize the effect of UC-MSC on monocyte-derived DC differentiation/maturation, we analyzed and compared the transcriptomes of purified iDC, mDC, iDC^MSC^ and mDC^MSC^ at the genome-wide level. The dendrogram of relationships after unbiased hierarchical clustering as well as principal component analysis (PCA) indicated that, irrespective of the donor, iDC^MSC^ and mDC^MSC^ had more similar phenotypes to each other than to iDCs and mDCs, which clustered together (Figure 3A, 3B), further corroborating the UC-MSC-induced modification of surface marker expression and cytokine secretion profiles. Comparison with publically-available expression profiling data (GEO dataset: GSE5099) showed that iDC^MSC^ (and mDC^MSC^ to a similar degree, see Figure S4) have a global transcriptional profile closer to that of M2-macrophages than to DC (Figure 3C). A more focussed analysis on classical macrophage (i.e. CD14, CD16, CD68) and M2-macrophage (i.e. CD163, IL10 and CXCR4 among others) gene transcripts [31] confirmed that in the presence of MSC, monocyte-to-DC differentiation was skewed towards M2-macrophages (Figure S5). Morphological studies (actin distribution, nuclear shape and cell size) by fluorescence microscopy confirmed that iDC^MSC^ cells were strikingly different in comparison to iDC. Indeed, iDC^MSC^ cells were smaller (3.43 times in average), with a cytoskeleton lacking the typical organization of filaments and focal adhesions (Figure 3D, 3E). Cellular protrusions were missing from iDC^MSC^ and mDC^MSC^. Moreover upon LPS treatment, mDC^MSC^ did not acquire the dendrites typical of mDC (Figure 3D). The size, shape and cytoplasmic appearance of both iDC^MSC^ and mDC^MSC^ were compatible with M2 macrophage morphology [32](Figure S6).

### UC-MSC skew monocyte-DC differentiation towards Th2 polarizing cells

**Figure 4:**
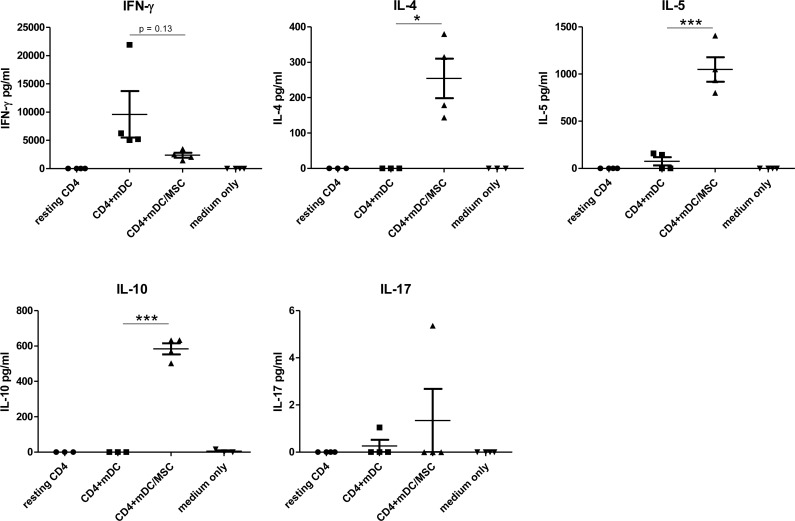
DC matured in presence of UC-MSC induce Th2 polarization of naïve CD4^+^ T-cells Peripheral blood monocytes were differentiated into mature DC in absence (mDC) or presence (mDC^MSC^) of UC-MSCs. We used primary UC-MSCs derived from 4 cord units. Naïve CD4^+^ T-cells were isolated from adult peripheral blood. 50.000 naïve CD4^+^ T-cells were cultured for 6 days with 20.000 allogeneic DCs (either mDC or mDC^MSC^, upon MSC removal) or left resting. After 6 days, 80.000 cells were re-stimulated with activating beads (Dynabeads Human T activator CD3/CD28 by Invitrogen) in X-VIVO 15 serum free medium in a final volume of 200 ml according to the manufacturer instructions (ratio cells:beads = 1:1). After 24 hours the cell supernatant was collected and tested for the presence of Th1 (IFNγ), Th2 (IL-4 and IL-5), Treg (IL-10) and Th17 (IL17) cytokines. *N* = 3 independent experiments. Statistics: unpaired t-test, 2 tails. *p < 0.05, ***p < 0.0001.

Since both transcriptome and morphological analysis indicated that the presence of UC-MSC during monocytes-DC differentiation resulted in the induction of cells similar to M2-macrophages, we tested the biological characteristics that distinguish these cells from regular *in vitro* differentiated DC. Although monocyte-derived DC as well as M1-macrophages are potent Th1 polarizing cells, M2-macrophages typically induce Th2 immune responses [26, 33]. To compare naïve T-cell polarization capacity, we cultured naïve CD4^+^ T-cells in the presence of allogeneic mDC or mDC^MSC^ for 6 days and then stimulated them with activating beads. We observed that mDC induced a Th1 cytokine secretion profile (IFNγ high, IL-4, IL-5 and IL-10 low), while mDC^MSC^ induced a Th2/regulatory profile (IFNγ low, IL-4, IL-5 and IL10 high) (Figure 4). We did not observe secretion of IL-17 in any of the conditions tested.

### Lactate secretion plays a key role in the alteration of DC differentiation induced by UC-MSC

**Figure 5:**
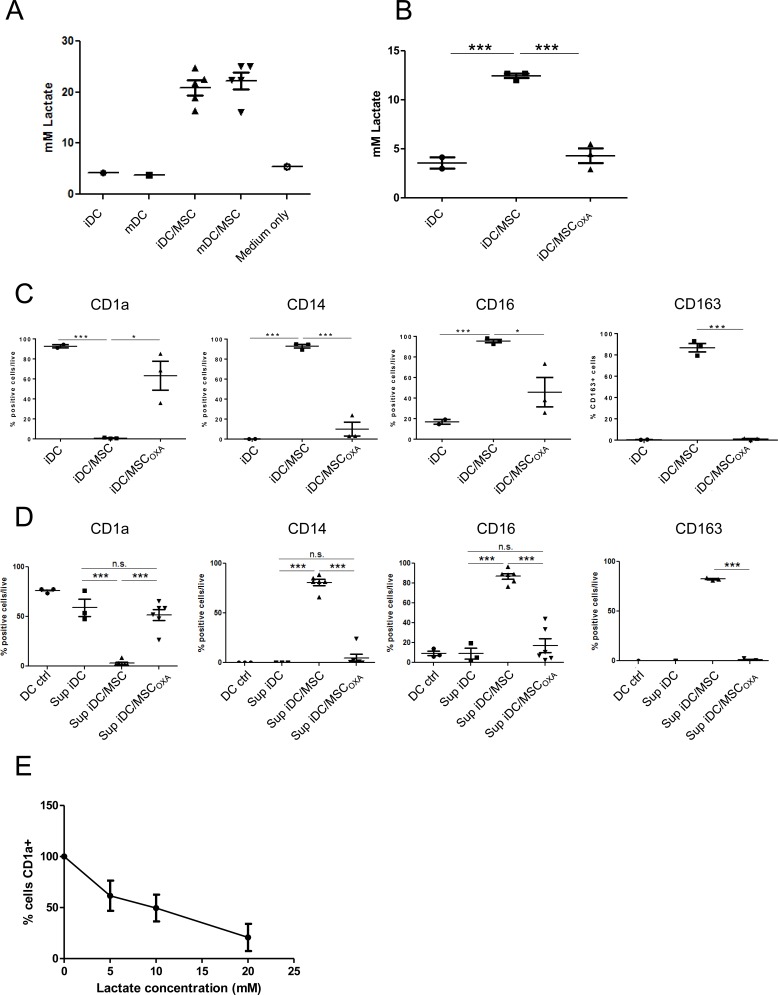
UC-MSC secrete high amount of lactate and inhibiting of lactate secretion eliminates their effect **A.** Lactate concentration was measured in cell culture supernatant of iDC and mDC obtained in presence or absence of UC-MSC. Measure was done at day 7 of culture. **B.** When UC-MSC were pre-treated for 1 hour with 2 mg/ml Oxamic acid (OXA) before starting the co-culture with monocytes, the presence of lactate in the supernatant was completely abolished. Data refer to culture medium harvested at day 2 of culture. UC-MSC source: A) UC-MSC cell line and 4 different primary UC-MSC. B) 3 different primary UC-MSC. **C.** Monocyte-DC differentiation was induced in presence or absence of primary UC-MSC. At the end of the culture the obtained cells were characterized by flow cytometry and CD marker expression was evaluated as percentage of living positive cells. When UC-MSC were pre-treated with OXA, 2 mg/ml for 1 hour before the beginning of the co-culture, their effect was severely decreased (*N* = 3). **D.** Monocytes were differentiated into DC in the presence or not of UC-MSC. Where indicated, UC-MSC were pre-treated 1 hour with 2 mg/ml OXA before starting the co-culture. After 48 hours, culture supernatants were collected and used to condition a second DC differentiation. After 3 days, cells were harvested and tested for CD marker expression. The percentage of living cells positive for each marker is reported. Conditioning the culture medium with supernatant obtained from co-cultures monocytes-UC-MSC was able to reproduce the effect of UC-MSC. Of note, OXA pre-treatment strongly blocks this effect. iDC ctrl refers to DC differentiated for 3 days in absence of conditioned medium. Statistics: unpaired t-test, *p < 0.05, **p < 0.01, ***p < 0.001. **E.** Monocytes were differentiated into DC in presence of Lactate at different concentrations. We report the percentage of CD1a+ cells normalized on the percentage found in the control condition (no Lactate treatment). Cells were analyzed at day 3 of culture. *N* = 3.

To determine if the M2-macrophage differentiating capacity of UC-MSC was mediated by the release of a soluble factor, we stimulated monocytes with GM-CSF and IL-4 in presence of UC-MSC separating the two cell types by a transwell system. As shown in Figure S7, surface marker modifications were similar with the changes observed when cells were in contact (Figure 1A). Culture medium was harvested from iDC and iDC^MSC^ cultures (day 2 of culture), diluted 1:2 with fresh medium containing GM-CSF and IL-4 and used to condition a second monocyte-into-DC differentiation culture. The medium collected from the iDC^MSC^culture was able to reproduce the effect of co-culture of monocytes with UC-MSC for both surface marker expression and cellular morphology (Figure S8A, B), while the medium obtained from the iDC culture did not alter the differentiation process (compare data referring to regular iDC and cells obtained by transferring iDC supernatant in Figure 5D). This confirms that the effect of UC-MSC is mediated by secreted factors.

It has previously been proposed that UC-MSC can affect immune cells through secretion of indoleamine 2,3-dioxygenase (IDO) or IL-6 [34–37]. We found that both IDO and IL-6 concentrations were significantly increased in the supernatant of UC-MSC/monocyte co-cultures (Figure 1B), in agreement with the hypothesis of a role played by these molecules in the UC-MSC effect. However, inhibiting their activity by using either 1-methyl-tryptophan or an IL-6 blocking antibody did not alter UC-MSC-mediated effect, indicating that neither IDO nor IL-6 played a key role in this process (not shown).

DC differentiation can be altered by the presence of high concentration of lactate, either endogenously produced or secreted by neighboring cells (i.e. in tumor microenvironment) [38, 39]. We observed that the presence of UC-MSC strongly induced lactate production (Figure 5A). The amount of lactate secretion by UC-MSC was independent of both the presence of cytokines and the contact between UC-MSC and monocytes (Figure S9), indicating that UC-MSC do not require a specific stimulus for lactate production. To evaluate the role of UC-MSC-secreted lactate in DC differentiation, we pre-treated UC-MSC for 1 hour with 2 mg/ml Oxamic acid (OXA, an inhibitor of the enzyme lactate dehydrogenase) before initiation of the co-culture of monocytes with UC-MSC. After 2 days of culture, lactate concentration was comparable to what was observed in the absence of UC-MSC (Figure 5B). Compared to iDC^MSC^, monocytes differentiated in the presence of UC-MSC pre-treated with OXA, showed a significant increase in CD1a expression and a significant decrease in the expression of CD14 and of the M2-macrophage markers CD16 and CD163 (Figure 5C). These findings indicate that OXA treatment impaired the effect of UC-MSC on iDCs. Moreover, medium collected from the co-culture of monocytes with OXA-pre-treated UC-MSC was not able to induce the iDC^MSC^ phenotype (Figure 5D). Exogenously added lactate during DC differentiation inhibited the acquisition of CD1a in a dose-dependent fashion (Figure 5E). In agreement with the ability of UC-MSC to secrete lactate independently of their contact with monocytes (Figure S9), we found that the medium of UC-MSC cultured alone in the presence of GM-CSF and IL-4 was able to alter the phenotype of monocyte-derived DC (Figure S8C). When MSC were cultured alone in a medium without cytokines, the effect was still reproduced, but to a lesser extent and proportionally to the amount of secreted lactate (not shown).

### UC-MSC alter the metabolism of differentiating DC

To retrieve relevant biological pathways associated with the effect of UC-MSC on monocyte-to-DC differentiation, we further analyzed the gene expression profile data by performing a functional classification analysis of differentially-expressed genes (FDR <= 0.01, Array signal>60) using the DAVID tool (version 6.7 [40, 41]) (Table 1). Focusing on the most highly-enriched functional terms between the transcriptome comparison of iDC with iDC^MSC^, we observed that genes related to mitochondrial function and oxidative phosphorylation had a strikingly lower representation in iDC^MSC^. Moreover, a query of mitochondrial proteome database [42] for the occurrence of mitochondrial gene products within the differentially-expressed [43] gene lists in iDC *versus* iDC^MSC^ transcript comparison confirmed that mitochondrial genes were underrepresented in iDC^MSC^ (Figure S10). This suggested the possibility that UC-MSC affect mitochondrion-related processes during the differentiation phase of monocytes into DC.

**Table 1:** Functional annotation analysis of differentially-expressed genes in iDC versus iDC^MSC^ transcriptome comparison

A) iDC-enriched/iDC^MSC^-depleted functional annotation terms								
Category	Term	Count	%	Fold Enrichment	PValue	Bonferroni	Benjamini	FDR
GOTERM BP FAT	GO:0055114^~^oxidation reduction	85	7.7	2.2	2.4E-12	6.9E-09	6.9E-09	4.3E-09
SP PIR KEYWORDS	oxidoreductase	76	6.9	2.3	1.1E-11	5.9E-09	5.9E-09	1.6E-08
GOTERM BP FAT	GO:0051186^~^cof actor metabolic process	39	3.5	3.3	9.8E-11	2.8E-07	1.4E-07	1.8E-07
GOTERM_BP_FAT	GO:0006732^~^coenzyme metabolic process	33	3.0	3.6	4.5E-10	1.3E-06	4.3E-07	8.0E-07
SP_PIR_KEYWORDS	acetylation	227	20.5	1.5	6.4E-10	3.5E-07	1.7E-07	9.3E-07
SP PIR KEYWORDS	Steroid biosynthesis	16	1.4	6.8	3.4E-09	1.9E-06	6.2E-07	5.0E-06
GOTERM CC FAT	60:0305739^~^mitochondrion	115	10.4	1.7	4.0E-09	1.9E-06	1.9E-06	5.7E-06
SP PIR_KEYWORDS	sterol biosynthesis	13	1.2	8.8	4.4E-09	2.4E-06	5.9E-07	6.4E-06
SP PIR KEYWORDS	mitochondrion	91	8.2	1.9	7.9E-09	4.3E·06	8.5E-07	1.1E-05
GOTERM CC FAT	GO:0031090^~^organelle membrane	114	10.3	1.7	9.8E-09	4.7E-06	2.3E-06	1.4E-05
GOTERM_BP_FAT	GO:0008610^~^1ipid biosynthetic process	47	4.2	2.5	2.0E-08	5.8E-05	1.5E-05	3.6E-05
KEGG_PATHWAY	hsa00100:Steroid biosynthesis	11	1.0	9.3	2.7E-08	4.7E-06	4.7E-06	3.3E-05
GOTERM BP FAT	GO:0016126^~^sterol biosynthetic process	14	1.3	6.6	5.9E-08	1.7E-04	3.4E-05	1.1E-04

B) iDC^MSC^-enriched/iDC-depleted functional annotation terms								
Category	Term	Count	%	Fold Enrichment	PValue	Bonferroni	Benjamini	FDR
GOTERM_BP FAT	GO:0006955^~^immune response	93	9.9	2.8	1.7E-19	4.6E-16	4.6E-16	2.9E-16
INTERPRO	IPR013783:Immunoglobulin-like fold	56	6.0	2.3	6.1E-09	6.9E-06	6.9E-06	9.8E-06
GOTERM BP FAT	GO:0006952^~^defense response	65	6.9	2.1	1.5E-08	4.1E-05	2.1E-05	2.6E-05
SP PIR KEYWORDS	Immunoglobulin domain	50	5.3	2.3	7.5E-08	3.8E-05	3.8E-05	1.1E-04
INTERPRO	IPRO07110:Immunoglobulin-like	50	5.3	2.3	7.7E-08	&7E-05	4.4E-05	1.2E-04
GOTERM BP FAT	GO:0009611^~^response to wounding	58	6.2	2.1	1.1E-07	3.1E-04	1.0E-04	2.0E-04
SP_PIR_KEYWORDS	immune response	31	3.3	3.0	1.9E-07	9.5E-05	4.8E-05	2.7E-04
GOTERM BP FAT	GO:0006954^~^inflammatory response	41	4.4	2.4	3.3E-07	9.2E-04	2.3E-04	5.9E-04
INTERPRO	IPR003599:Immunoglobulin subtype	39	4.2	2.4	7.7E-07	&7E-04	2.9E-04	1.2E-03
SMART	5M00409:1G	39	4.2	2.2	3.4E-06	8.5E-04	8.5E-04	4.4E-03
KEGG PATHWAY	hsa04060:Cytokine-cytokine receptor interaction	35	3.7	2.3	5.0E-06	7.5E-04	7.5E-04	5.9E-03
GOTERM MF FAT	60:0003823^~^antigen binding	11	1.2	6.2	6.4E-06	5.0E-03	5.0E-03	9.8E-03
INTERPRO	IPR013106:Immunoglobulin V-set	26	2.8	2.6	1.8E-05	2.0E-02	5.2E-03	2.9E-02

Lists of non-redundant differentially-expressed genes (FDR≤0.01, Array signal>60) were submitted to DAVID tool and queried for enrichment of functional terms compared to a background list of genes found in Agilent Human Genome arrays. (A) The list of 13 highly significant functional terms, for a total of 1108 iDC-enriched gene identifiers, are displayed. Mitochondrion-related processed are overrepresented. (B) The list of 13 highly significant functional terms, for a total of 938 iDC-enriched gene identifiers, are displayed. Genes related to immune response are overrepresented.

**Figure 6:**
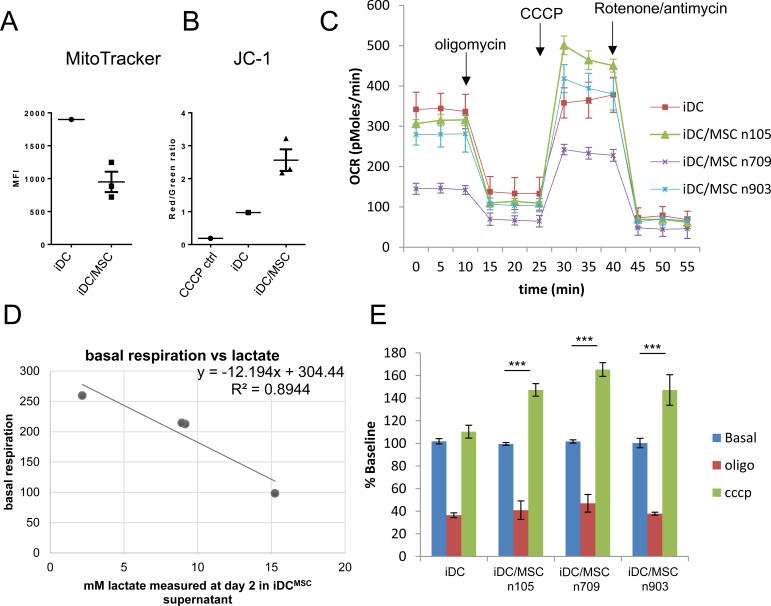
UC-MSC alter the metabolism of monocytes differentiating into DC Monocytes were differentiated into immature DC in presence or absence of UC-MSC. At the end of the differentiation (day5), mitochondrial mass and mitochondrial membrane potential were assessed by MitoTracker (A) and JC-1 (B) staining by flow cytometry (UC-MSC were excluded from the analysis based on their higher physical parameters). The ratio between the red and green fluorescence of JC1 is proportional to the potential across the mitochondrial membrane. In both **A.** and **B.** we tested 3 different primary UC-MSC. **C.** Monocytes were stimulated to differentiate into DC in presence of medium conditioned by a previous culture in which monocytes were differentiated into DC in presence or absence of UC-MSC (iDC and iDC^MSC^, supernatant collected at day 2). At day 3 cells were harvested and oxygen consumption rate (OCR) was measured at the basal level and in response to specific drugs with a Seahorse instrument. Three primary UC-MSC were used for the experiment and the basal OCR of the tested cultures was proportional to the amount of lactate present in the medium conditioned by UC-MSC **D. E.** We report the variation of OCR normalized on the basal consumption to put in evidence the variation in spare respiratory capacity induced by the medium conditioned by UC-MSC. Statistics: t-test, ***p≤0.001. The numbers 105, 709 and 903 refer to the ID of the 3 primary UC-MSC used for the experiment. All experiments have been independently performed with two different normal donors in response to the 3 primary UC-MSC with consistent results. Bars in C and E indicate standard deviation.

Metabolic reprogramming and consequent increase or decrease in the activity and number of mitochondria is a physiological requirement for DC differentiation and activation [44, 45]. The high concentration of lactate (a by-product of glycolysis) produced by UC-MSC in iDC^MSC^ cultures together with the microarray pathway analysis suggested that a metabolic alteration could be responsible for the altered differentiation of the monocytes into DC. We observed that in presence of UC-MSC, differentiating DC showed a decreased mitochondrial mass, as revealed by MitoTracker staining (Figure 6A). When stained with JC-1, a sensor of mitochondrial polarization that changes the wavelength of emission according to the polarization of the organelle's membrane turning from red (polarized) to green (depolarized), iDC^MSC^ displayed higher ratio polarized/depolarized mitochondrial membrane (Figure 6B).

Because the membrane polarization is fundamental for mitochondrial ATP generation, a higher JC-1 polarized/depolarized ratio could mean either an increase in mitochondrial function or a reduction of oxidative phosphorylation with consequent accumulation of the membrane potential. To test this hypothesis, we measured the oxygen consumption rate during the differentiation of monocytes into DC in presence of medium collected from either iDC or iDC^MSC^. After 3 days of culture, cells were harvested and live oxygen consumption rate (OCR) was measured at baseline and in response to oligomycin, cyanide 3-chlorophenylhydrazone (CCCP) and rotenone/antimycin with a Seahorse analyzer. The OCR in response to CCCP indicates the maximal mitochondrial activity while the difference between the basal OCR and the OCR in response to CCCP is a measure of the spare respiratory capacity. As shown in Figure 6C, the presence of medium collected at day 2 from iDC^MSC^ cultures lowered basal OCR. This effect was proportional to the lactate concentration measured in the conditioning medium (Figure 6D). Upon oligomycin treatment, the drop in OCR, evaluated as percentage of basal respiration, was comparable across the different treatments, indicating that ATP turnover (basal mitochondrial OCR used for ATP synthesis) and the coupling efficiency were not significantly affected (Figure 6E). Cells treated with medium collected form iDC^MSC^ showed a considerably higher spare respiratory capacity (see green bar in Figure 6E). These observations are in accordance with data obtained with both MitoTracker and JC-1, as they show that cells treated with iDC^MSC^ medium have lower basal respiration but more polarized mitochondrial membrane potential. Taken together, these data indicate that the medium harvested form an MSC-monocyte co-culture in the presence of GM-CSF and IL-4 altered the metabolic properties of monocytes differentiating into DC. Both the lower OCR and the higher spare respiratory capacity, compared to monocyte-derived DC obtained in absence of MSC, are compatible with an M2-macrophage metabolic profile [46].

## DISCUSSION

In the present work, we investigated the effect of UC-MSC on monocyte-derived DC differentiation. We show for the first time that human UC-MSC divert the differentiation of human monocytes from DC towards M2-macrophages by metabolic reprogramming through lactate secretion. These data suggest that MSC may induce immunomodulation by supporting M2-macrophage differentiation, as M2-macrophages have been reported to have anti-inflammatory, tissue repair-inducing properties [26]. Despite the difficulty of tracking intravenously injected MSC *in vivo* [47], several line of evidence indicate that MSC, as well as monocytes, are recruited to the site of inflammation [2, 48, 49] suggesting the possibility of interactions between these two cell types in areas of tissue damage or infection.

Although it has been shown that MSC interfere with human monocyte-derived DC differentiation [50] and several soluble factors (i.e. IDO, IL-6, PGE-2, GRO-γ) have been proposed as mediators [36, 51–54], this is the first time that the metabolic reprogramming ability of MSC through lactate secretion is highlighted as a mechanism of action in MSC-induced immunomodulation. Previous reports on the MSC-mediated M2 polarizing effect are scarce. It has previously been shown that co-culture of human peripheral blood mononuclear cells with bone marrow-derived MSC (BM-MSCs) stimulated by interferon-gamma induced a small fraction of monocytes to differentiate into M2-like macrophages [37] based on the acquisition of the CD206 surface marker and IL-10 expression. Our study not only confirms these findings by using CD163, which is another M2-macrophage surface marker, but also provides a much more extensive analysis of the cells obtained in presence of MSC focusing on both phenotypical and functional properties. In our experimental conditions, by differentiating monocytes into DC with GM-CSF and IL-4, we showed that cells differentiated in presence of UC-MSCs exhibited membrane antigen expression (CD1a-, CD80^low^, CD83^low^, CD86^+^, HLA-DR^+^, CD16^+^, CD163^+^), morphology, migration properties, antigen uptake capacity, gene expression profile, Th2 polarizing ability and metabolic profile that are all characteristic of or compatible with an M2-macrophage phenotype [26].

We propose lactate secretion as an important mediator of the effect of UC-MSC. Indeed, pre-treating UC-MSC with a lactate dehydrogenase inhibitor (oxamic acid) before their co-culture with monocytes, decreased the concentration of lactate in the supernatant and inhibited the UC-MSC effect. The abrogation of the effect of UC-MSC by oxamic acid pre-treatment was observed both in MSC-monocyte co-cultures and upon transfer of iDC^MSC^ supernatant. Interestingly, it has been shown that cancer cells are able to polarize DC towards tolerogenic cells by poisoning the tumour microenvironment with high amount of lactate [39, 55], indicating that modification of local lactate concentration is a signal tuning immune response. Because we found that UC-MSC secrete high amount of lactate, it is tempting to postulate that they can induce immune response inhibition *in vivo* by the same mechanism. Importantly, it has been shown that, when cultured *in vitro* at high cellular density, monocytes were not able to differentiate into DC and failed to produce IL-12 due to the accumulation of lactate in the culture medium [38]. In agreement with these findings, we observed that, following exogenous administration of lactate during DC differentiation (in absence of UC-MSC), the efficiency of the differentiation process was inversely correlated with the lactate concentration in the culture medium.

**Figure 7:**
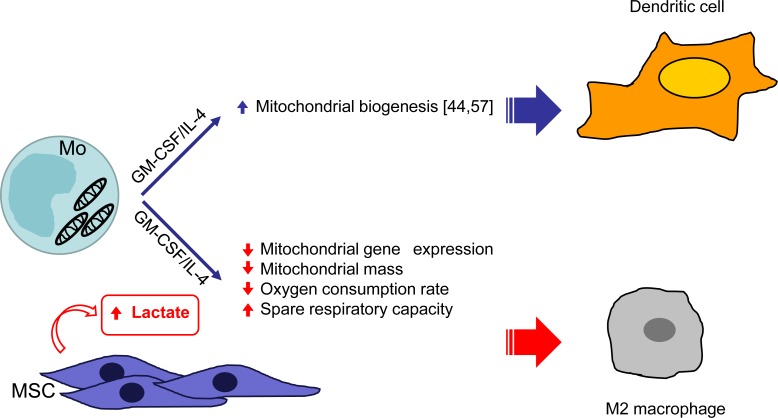
schematic representation of the effect of UC-MSC on the metabolism of monocyte-derived DC inducing M2 macrophages

By interrogating our microarray data, we observed that in presence of UC-MSC, cells modulated the expression of genes related to energetic metabolism. It is well documented that immune cells undergo metabolic switches to elicit specific functions [46]. For example, DC maturation is accompanied by increased glycolysis [56]. The pivotal role of cell metabolism in DC differentiation has been previously demonstrated by showing that monocytes differentiating into DC undertake active mitochondrial biogenesis and that treatment with rotenone inhibits differentiation by blocking oxidative phosphorylation [44, 57]. Further pathway analysis of our data revealed that, in comparison to iDC, iDC^MSC^ down-regulated genes that are associated with mitochondrial activity and oxidative phosphorylation. BM-MSC have been proposed to promote cancer cell survival in the presence of pro-apoptotic treatment by regulating mitochondrial activity and fostering the Warburg effect [58]. In our setting, the analysis of mitochondria by MitoTracker and JC-1 staining revealed that cells differentiated in the presence of UC-MSC presented lower mitochondrial mass but more polarized mitochondrial membrane potential. These data were confirmed by real time analysis of the OCR in response to different drugs targeting mitochondrial function. The basal OCR was lower in cells treated with iDC^MSC^-conditioned medium and directly proportional to the lactate produced by the primary UC-MSC, which is in agreement with the lower MitoTracker mean fluorescent intensity. The lower OCR and higher spare respiratory capacity detected in cells treated with iDC^MSC^-conditioned medium suggest that these cells are not relying as much on oxidative phosphorylation to meet their energetic needs, but are primed to promptly respond to stimuli, consistent with what has been shown for M2-macrophages [59]. Based on our data, we propose that UC-MSC-secreted lactate modifies the mitochondrial activity of differentiating cells and induces a skew towards M2-macrophages by metabolic reprogramming (depicted in Figure 7).

The detailed mechanism(s) by which UC-MSC-secreted lactate can achieve the observed metabolic alterations remain unknown. Based on current knowledge, exogenous lactate can modify the cellular homeostasis either by directly trafficking through monocaboxylic transporters (MCTs) or by triggering a specific receptor, HCAR1. In the former case, the molecule physically moves across the plasma membrane, while in the latter, it triggers a yet uncharacterized intracellular pathway. It will be interesting to study the pathway by which UC-MSC-secreted lactate induces these modifications in differentiating monocytes and the link with mitochondrial metabolism. Lactate agonists have been shown to cure lipolysis in both mice and humans without generating cutaneous flushing [60, 61]. It has been suggested that Langerhans cells, that are responsible for the flushing, are sensitive to HCAR1 stimulation, thereby directly connecting the effect of lactate with antigen presenting cell activity [62]. Moreover, in mice, lactate decreases liver and pancreatic damage by innate immunity inhibition [63]. These reports are in agreement with an immunomodulatory function of lactate *in vivo*. The idea of using lactate secretion as a tool for locally tuning myeloid cell plasticity to achieve immune regulation opens interesting avenues from a translational point of view. The feasibility of such an approach has been successfully demonstrated for human cancers [64]. Finally, the observation that the amount of lactate produced by the different primary UC-MSC was donor-dependent could be further explored for the development of biomarkers predicting the efficacy of UC-MSC as immunomodulators.

In summary, we demonstrated that the presence of UC-MSC during the GM-CSF/IL-4-induced differentiation of human monocytes, skewed the differentiation towards M2 macrophages instead of DC. This effect was largely due to MSC-secreted lactate that induced metabolic modifications. Our findings could have potential clinical implications considering that MSC cell therapy could be replaced/accompanied by drugs targeting metabolic modulation by lactate.

## MATERIALS AND METHODS

### Human samples

Human peripheral blood mononuclear cells were obtained by Ficoll-Hypaque separation from peripheral blood or leukapheresis from healthy donors following informed written consent approved by ethical committee.

### Chemicals

Oxamic acid (O3750) and L-(+)-Lactate (L6402) were from Sigma Aldrich, St. Louis, MO.

### UC-MSC isolation and culture

Primary UC-MSC were obtained cells from 4 different cord units upon collagenase digestion. Stromal cells were tested by flow cytometry as CD44^+^, CD31^−^, CD105^+^, CD90^+^, CD45^−^ upon gating on live cells (7AAD^−^). Unless specified in the figure legend, all the presented data refer to primary UC-MSC, either isolated form umbilical cords in our Institution or kindly provided by Hema-Québec. The latter were certified for both marker expression and tri-lineage differentiation capacity. In both cases informed consent from the mothers was obtained. For experiments requiring a large number of cells we used immortalized human cord-blood MSC as previously described [10, 65].

### DC differentiation and maturation

Monocytes were isolated by CD14 positive selection (Stemcell Technology, Vancouver, BC, Canada) (purity above 95%). Cells (5×10^5^/mL) were cultured for 5 days in the presence of human recombinant granulocyte-macrophage colony-stimulating factor (GM-CSF) (50 ng/mL; R&D Systems, Minneapolis, MN) and IL-4 (10 ng/mL; eBioscience, San Diego, CA) to obtain immature DCs (iDCs). Mature DCs (mDCs) were obtained by stimulating iDCs with lipopolysaccharides (LPS) (1 μg/mL; Sigma, St Louis, MO) for 2 additional days, as previously described [65]. Every 2-3 days, half of the medium was replaced by fresh medium with cytokines. UC-MSC (ratio MSC:monocytes = 1:5) were plated during the phase of DC differentiation and maturation according to the scheme reported in Figure S1. In experiments requiring co-culture of monocytes and MSC without physical contact, cells were separated by a transwell system. Briefly, 2×10^5^ monocytes were plated in the bottom chamber of a 24 well plate, separated from MSC (4×10^4^) by a 0.4 μm porous membrane (Corning, Tewksbury, MA). When needed (i.e. for DC mRNA extraction) UC-MSC were depleted from the culture based on their adhesive properties and purity of the obtained cells was tested by flow cytometry (above 95% of cells were CD45^+^). The phenotype of monocytes, immature DC (iDC) and mature DC (mDC) was tested by flow cytometry as previously described [65].

### Immunofluorescence

Cells were plated on poly-lysine treated coverslips, fixed and permeabilized (Cytofix-cytoperm kit, BD Bioscience). Actin filaments were visualized with Phalloidin-Alexa488 (Life Technologies, Burlington, Canada) and nuclei with DAPI. Cells were analyzed with Olympus BX51 fluorescence microscope equipped with a Retiga 2000R camera (QImaging, Surrey, Canada). Overlays were obtained using Photoshop software. Area analysis was performed by using the free software ImageJ available at http://imagej.nih.gov/ij/index.html.

### Cytokine production analysis

Cytokines in cell culture supernatants were analyzed by Bio-Plex Pro Human Cytokine 17-plex Assay (Bio-Rad Laboratories, Mississauga, Canada) according to the manufacturer's recommendation. Results were analyzed on a CS 1000 Autoplex Analyzer (Perkin Elmer Inc., Waltham, MA). TGFβ was detected by ELISA (R&D Systems).

IDO quantification

The enzyme IDO catalyzes the degradation of the essential amino acid L-tryptophan to N-formylkynurenine, which can be quantified in a colorimetric assay as described [66]. Briefly, supernatant was diluted with as solution of 30% trichloracetic acid (ratio 2:1), and incubated 30 minutes at 50°C. Samples were spin 1 minute at 15.400g and the supernatant was collected and diluted 1:1 with Ehrlich's reagent. Absorbance at 490 nm was detected with an ELISA plate reader and control wells values subtracted.

### *In vitro* migration assay

25.000 mature DC obtained or not in presence of UC-MSC were plated on top of a 5 μm pores filter (Neuroprobe ChemoTx^®^system) and allowed to migrate for 2 hours at 37°C towards either medium only (RPMI medium containing 0.2% BSA) or attractant chemokines (CCL19, 300 ng/ml, CCL21, 500 ng/ml, both from PeproTech). Cells remaining in the upper compartment were gently removed, the plate was centrifuged and migrated cells were manually counted.

### *In vivo* migration assay

To test the migratory properties of mature human DC *in vivo*, we referred to the model described by Zhang et al [30]. 2 x10^6^ cells (either mDC or mDC^MSC^) were injected in the footpad of anesthetized C57/B17 SCID mice (Charles River). After 24 hours mice were anesthetized and foot pads were injected with 30 μl Evan's blue (0.1% in PBS). After 10 minutes animals were euthanized, poplitear lymph nodes were surgically removed and fixed in 3.7% formalin. Lymph nodes were paraffin embedded and 5 μm sections were stained with anti-human CD45 (DAKO, Burlington, Canada). The protocol has been approved by the Sainte-Justine Hospital's ethical committee for animal experimentation (CIBPAR).

### Antigen up-take assay

iDC and iDC/MSC (10^5^ cells/test) were incubated for 90 minutes at 37°C with pHRodo Green Zymosan-coated beads (ThermoFisher Scientific, Burlington, ON) according to the manufacturer's instructions. As negative control, cells were pre-incubated with 2 μg/ml Cytochalasin-B (Sigma Aldrich), an inhibitor of the engulfment process. Cells were analyzed by flow cytometry and frequency of internalized beads was calculated.

### Expression profiling analysis

Total RNA was extracted from dendritic cell samples using QIAzol Lysis Reagent (Qiagen) and purified using mRNA Easy Kit (Qiagen, Valencia, CA). RNA concentration was measured with Nano Drop ND-1000 Spectrophotometer (Nano Drop Technologies, Wilmington, USA) and its quality assessed with an Agilent 2100 Bioanalyser (Agilent Technologies, Waldbronn, Germany). High quality RNA from samples as well as Universal Human Reference RNA (Stratagene, Santa Clara, CA) were separately labeled with Cy5 and Cy3, respectively, using LowInput QiuckAmp Labeling Kit (Agilent Technologies, Santa Clara, CA). These were hybridized together onto Agilent Chip Whole Human genome, 4×44K (Agilent Technologies) according to the manufacturer recommendations. After analysis of the two-color array images by Agilent's Feature Extraction software, raw array data was pre-processed and normalized using FlexArray software (Genome Quebec, Canada) and the Bioconductor package. Simple Loess normalization was performed without background correction of the raw data after which differential expression and statistical significance were assessed using the “limma” algorithm [67]. More stringent correction of the resulting p-values was done by calculating the false discovery rate (FDR) according to the Benjamini-Hochberg method [68]. In order to compare expression profiling data obtained from different platforms, we used the DWD algorithm [69] for cross-platform normalization prior to hierarchical clustering and PCA analysis, which were done using Genesis software [70]. Microarray data used in this study are available at the Gene Expression Omnibus (GEO, http://www.ncbi.nlm.nih.gov/geo/) repository (GSE68962).

### Th1/Th2/Th17 polarization analysis

Naïve CD4^+^ T-cells were isolated by sorting CD25- CD45RO^−^ cells with a BD FacsAria cell-sorter upon CD4 enrichment. The purity of the collected naïve cells was above 95%. 50.000 naïve CD4^+^ T-cells were cultured in presence of 20.000 DC (either mDC or mDC^MSC^) or left unstimulated. MSC were depleted from the mDC^MSC^ culture as described in the methods section. At day 6 cells were harvested, counted, and 80.000 cells were re-stimulated for 24 hours with activating beads (Dynabeads Human T activator CD3/CD28 by Invitrogen) in X-VIVO 15 serum free medium in a final volume of 200 μl according to the manufacturer instructions. Secreted IFNγ, IL-4, IL-10, IL-5 and IL-17 were quantified by ELISA (IFNγ and IL-4 ELISA kits were from Peprotech, IL-10 and IL-17 from eBioscience, IL-5 from R&D Systems). Experiments were done in triplicate using the cord-blood derived MSC cell line and 4 times with primary UC-MSC derived from 4 different cord units.

### Lactate measurement

Secreted lactate was measured using either the Glycolysis Cell-Based Assay Kit by Cayman or an Architect c8000 analyzer (Abbott, Abbott Park, Illinois, USA).

### Mitochondria analysis

Mitochondria were quantified by staining the cells with MitoTracker^®^ Deep Red FM (Life Technologies). Mitochondrial polarization was measured by staining the cells with JC-1 (5,5′,6,6′-tetrachloro-1,1′,3,3′-tetraethylbenzimidazolylcarbocyanine iodide, Invitrogen). Cells were analyzed by flow cytometry.

### Metabolism analysis

Medium was harvested at day 2 from either iDC or iDC^MSC^ cultures, tested for lactate concentration and used for conditioning a new monocytes-DC differentiation. After 3 days cells were harvested and plated in a poly-lysine treated Seahorse 24well plate (200.000 cells/well). The rate of oxygen consumption deriving from mitochondrial OXPHOS was assessed using an extracellular flux analyzer (Seahorse Biosciences) according to manufacturer recommendations. After measuring basal respiration, ATP-linked OCR (ATP turnover) were determined by injecting oligomycin (1 μM). CCCP, an uncoupler of the electron transport chain, was used at a concentration of 1.5 μM to determine the maximal respiration rate. Rotenone (1 μM), an inhibitor of Complex I, and Antimycin A (1 μM), an inhibitor of Complex III, were used to completely inhibit mitochondrial electron transport to determine non-mitochondrial oxygen consumption. Mitochondrial basal respiration, ATP turnover, and the maximal respiration were calculated after correcting for the non-mitochondrial OCR.

### Statistics

Results were analyzed using GraphPad Prism version 5.0 (GraphPad Software Inc., La Jolla, CA). Error bars indicate standard error mean, unless specified. To compare different conditions unpaired t-test was used. Significance was set at p ≤ 0.05.

## SUPPLEMENTARY MATERIAL FIGURES


