# Supplementary material for: Human mesenchymal stromal cell-secreted lactate induces M2-macrophage differentiation by metabolic reprogramming
Source: Oncotarget. 2016 Apr 6;7(21):30193–210. doi: 10.18632/oncotarget.8623 (PMC5058674; doi:10.18632/oncotarget.8623)
Supplement: Supplementary file 1 [file oncotarget-07-30193-s001.pdf]

Human mesenchymal stromal cell-secreted lactate induces M2-macrophage differentiation by metabolic reprogramming

Supplementary Material

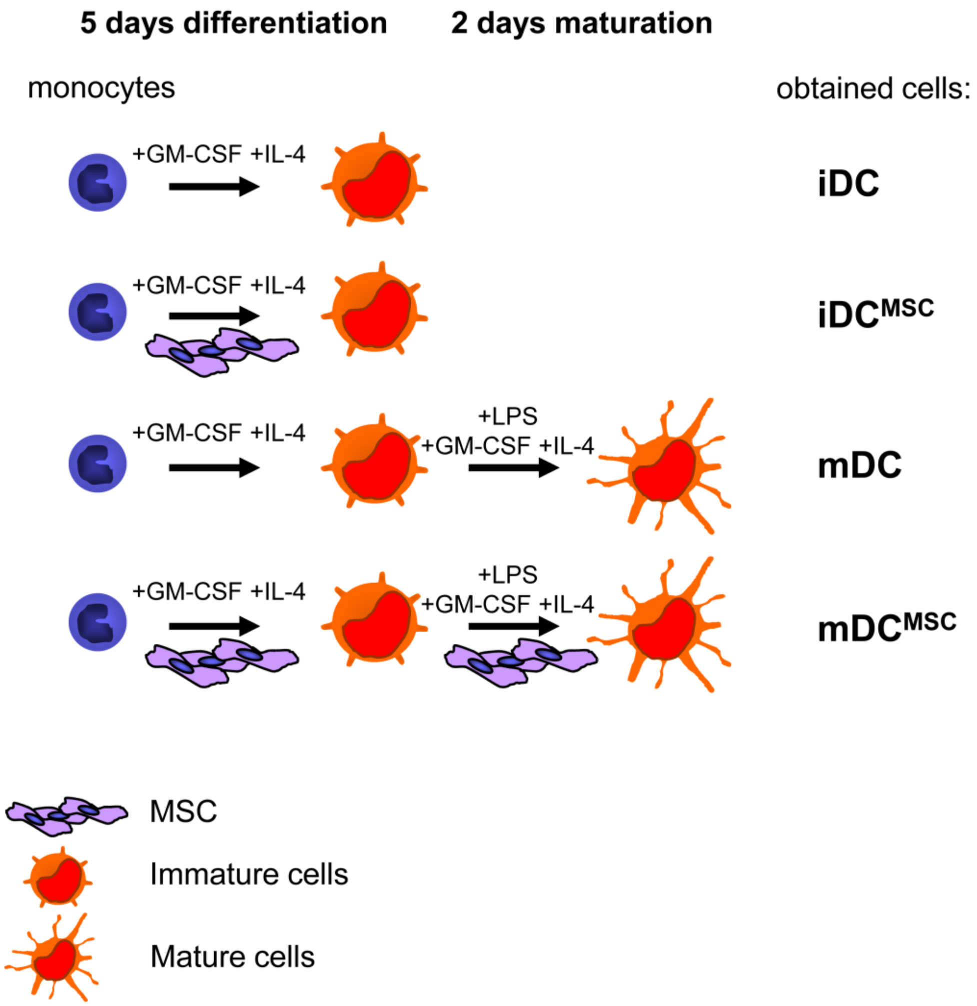

Supplementary Figure 1: Visual representation of the treatments leading to the 4 different types of DCs studied. Immature DC were produced in the absence (iDC) or presence (iDC^{MSC}) of UC-MSC. mDC refers to mature DC produced in absence of UC-MSC. mDC^{MSC} refers to cells co-cultured with UC-MSC during both the differentiation and maturation phases.

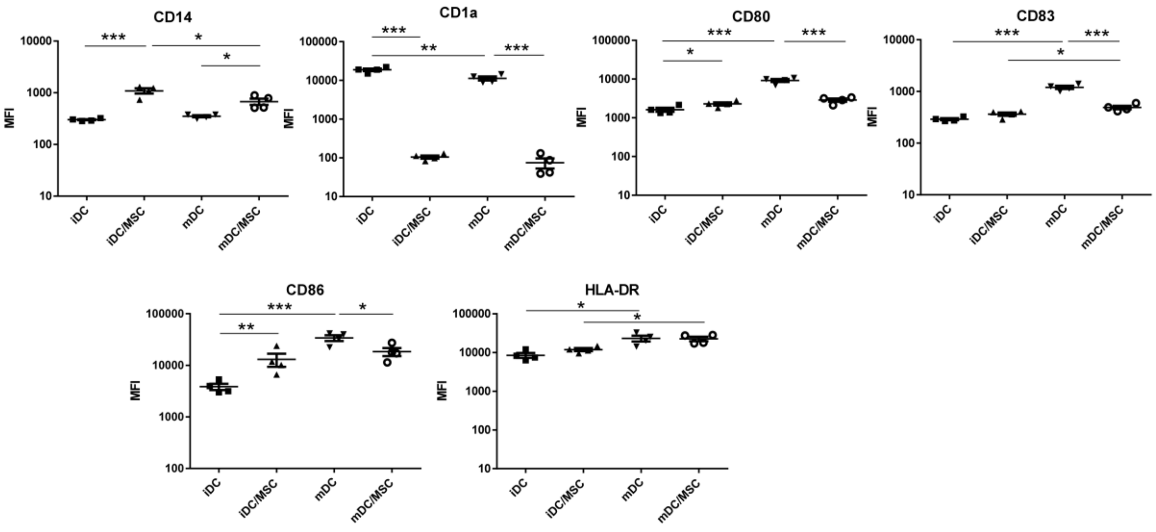

Supplementary Figure 2: DC Surface marker expression in presence or absence of UC-MSC cell line. At the end of the culture (day 7), cells were harvested and surface markers were analyzed by flow cytometry. The expression of CD1a (DC marker), CD80 and CD86 (co-stimulatory molecules), HLA-DR (important for antigen presentation) and CD83 (maturation marker) were assessed gating on DC based on their FSC/SSC parameters. Statistics: unpaired t-test, *p<0.05, **p<0.01, ***p<0.0001. iDC were compared with iDC^{MSC} and mDC, iDC^{MSC} with mDC^{MSC}, mDC with mDC^{MSC}. MFI: mean fluorescent intensity. N=4

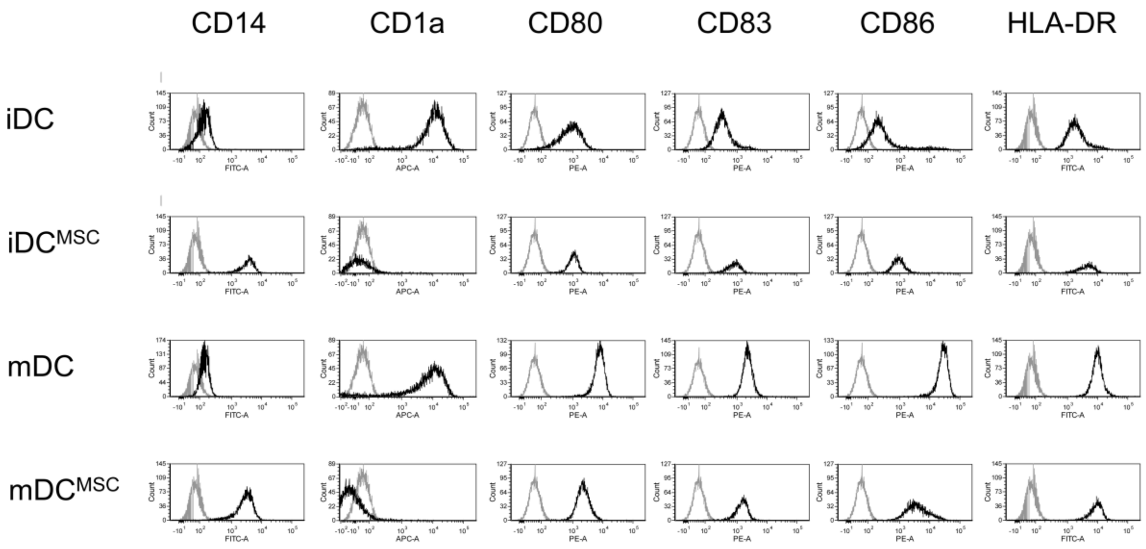

Supplementary Figure 3: surface marker expression of differentially obtained DC. At the end of the culture cells were harvested and surface markers were analyzed by flow cytometry. We considered the expression of CD1a (DC marker), CD80 and CD86 (co-stimulatory molecules), HLA-DR (important for antigen presentation) and CD83 (maturation marker). Data of a representative experiment are reported. MSC: primary UC-MSC. Data were collected upon gating on DC.

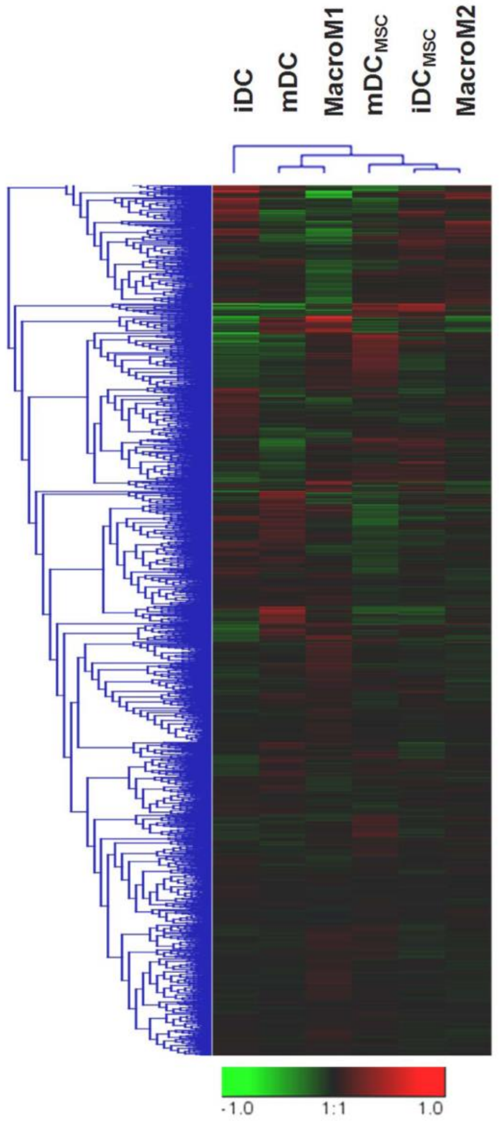

Supplementary Figure 4: the presence of UC-MSC induces an M2-macrophage signature in monocytes differentiating into DC. Monocytes were differentiated into iDC and matured into mDC in presence or absence of MSC. The heatmap shows that cells obtained in presence of MSC (both immature and mature) cluster together with M2-macrophages. MSC were physically removed from the culture before RNA was extracted from DC.

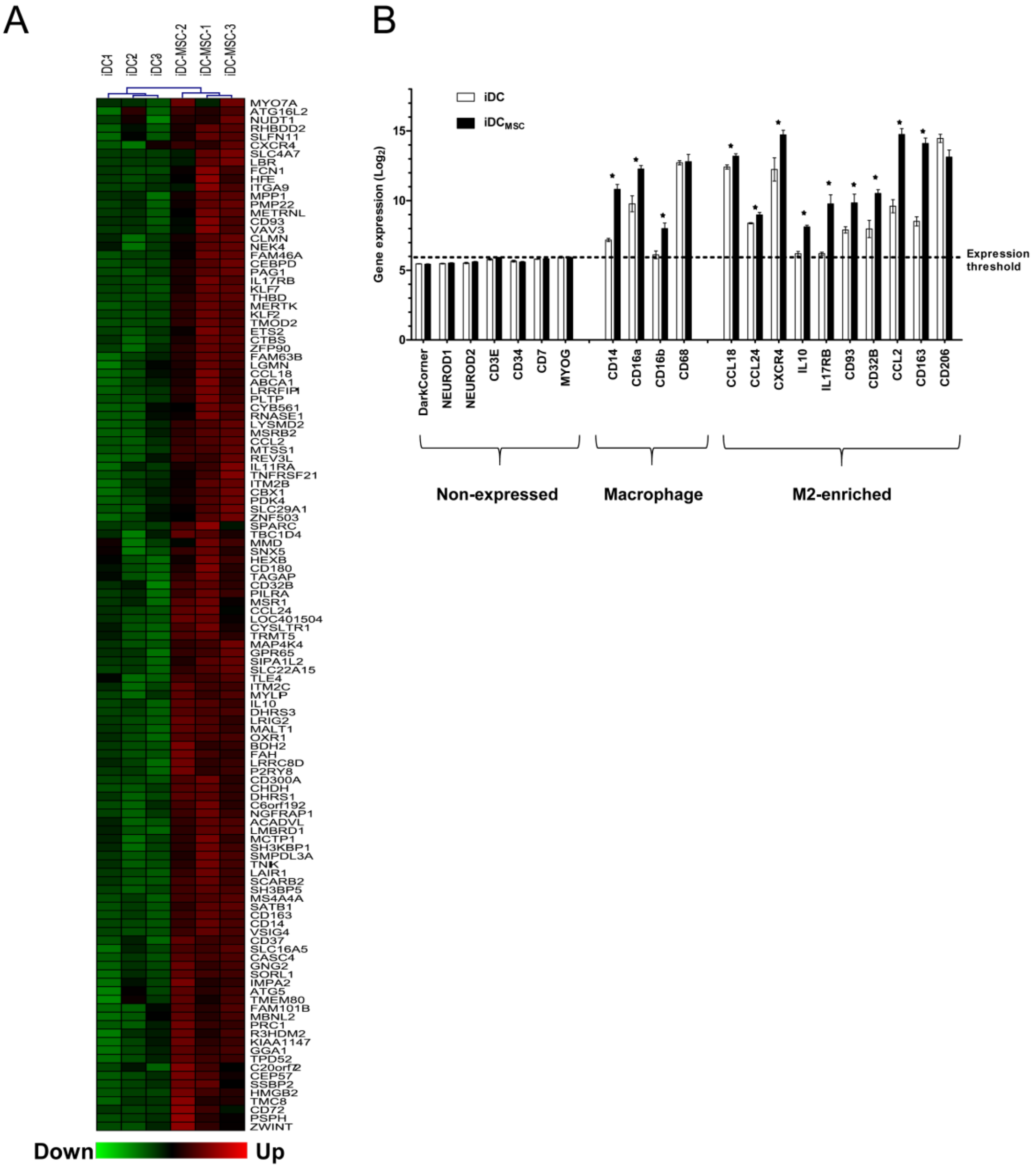

Supplementary Figure 5: iDC^{MSC} up-regulate the expression of genes associated with M2 macrophage profile. A) Heat map of relative expression of 122 M2 macrophage-enriched RNA transcripts [31] between iDC and iDC^{MSC} cells (n=3 donors). Red signifies an enrichment and green signifies a depletion for a particular transcript in iDC versus iDC-MSC comparison. All of these transcripts are significantly enriched (false-discovery rate ≤ 0.05) in iDC-MSC cells

compared to iDC cells. B) RMA-normalized microarray expression values (mean +/- SEM, n= 3 donors) for selected gene transcripts in iDC and iDC^{MSC} samples. Genes were chosen and grouped based on their non-expression in myeloid cells, their classical expression in macrophages and their well-described enrichment in M2-polarized macrophages [31, 71-76]. Asterisk (*) denotes gene probes that significantly (FDR ≤ 0.05) vary between iDC and iDC-MSC samples. A gene expression threshold value (discontinuous line) was established based on non-expressed genes and control probes (DarkCorner).

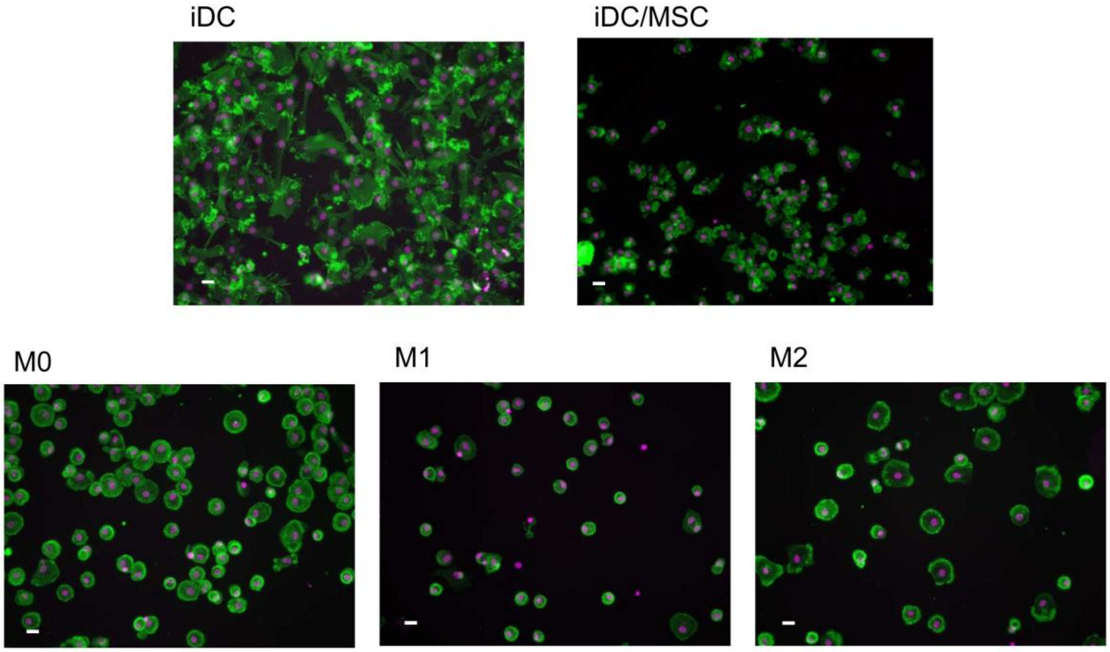

Supplementary Figure 6: morphology of monocyte-derived macrophages stimulated in M1 and M2 skewing conditions. Monocytes were differentiated in presence (iDC^{MSC}) or absence (iDC) of UC-MSC. Monocytes-derived macrophages (M0) were obtained by culturing monocytes for 5 days in presence of GM-CSF (50 ng/ml). To induce the differentiation of M1 macrophages the cells were stimulated for an additional 24h with IFNγ (20 ng/ml) and LPS (100 ng/ml). To induce M2 differentiation cells were stimulated for 24h with IL-4 (20 ng/ml). At the end of the stimulation cells were harvested with cold PBS and plated on polylysine-treated coverslips for fluorescence microscopy analysis. Green: actin; pink: nuclei. Bar: 10 μm.

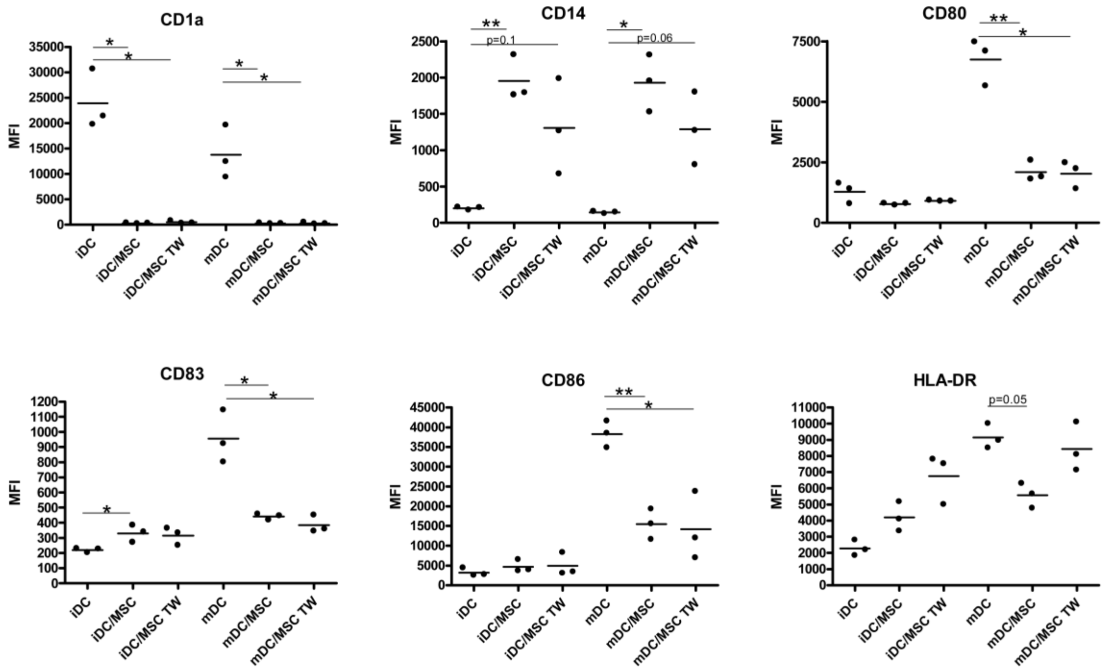

Supplementary Figure 7: UC-MSC alter DC differentiation in both contact and transwell cell culture conditions. Monocytes were plated alone, in contact with UC-MSC or in presence of UC-MSC but separated by a transwell system, and stimulated with GM-CSF and IL-4, matured or not with LPS as described. UC-MSC were able to alter DC differentiation and maturation in both contact and transwell conditions. Statistics: paired t-test, 2 tails, $*p<0.05$; $**p<0.01$.

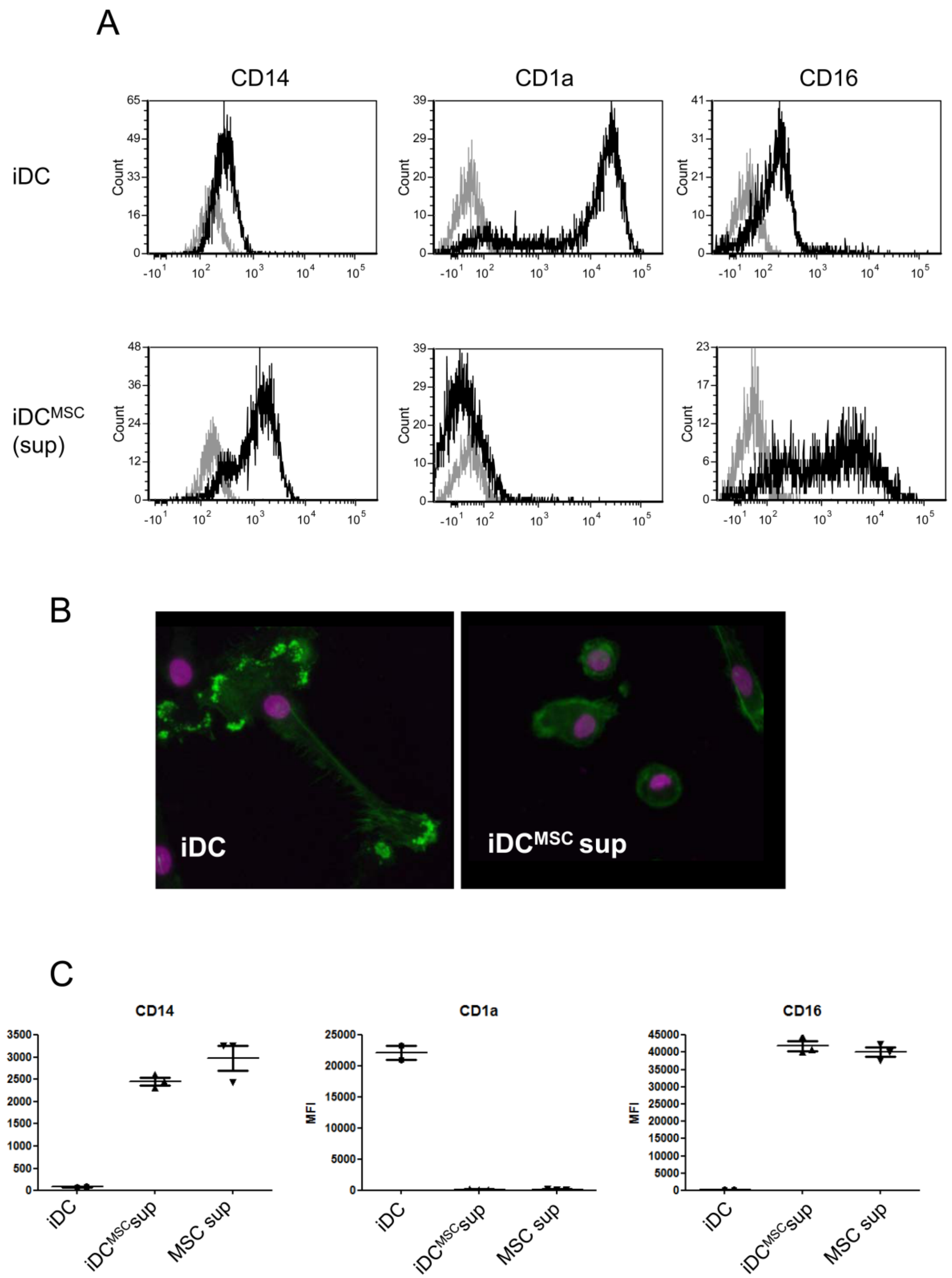
A
CD14
CD1a
CD16
iDC
iDC^MSC (sup)
Count
B
iDC
iDC^MSC sup
C
CD14
CD1a
CD16
MFI
iDC
iDC^MSC sup
MSC sup

Supplementary Figure 8: monocytes were differentiated into DC in presence or absence of UC-MSC. At day 2 of culture medium was collected and used to condition a new differentiation. After 2-3 days half of the medium was replaced with new conditioned medium. At day 5 cells from this second culture were harvested and evaluated in terms of surface marker expression (A) and morphology (B). The same pattern of CD marker expression could be obtained also by conditioning the new culture with medium harvested from MSC cultured alone in presence of GM-CSF and IL-4 (C). iDC: monocyte-derived immature DC; iDC^{MSC} sup: monocytes cultured in DC differentiating medium in presence of supernatant collected form iDC^{MSC} culture; MSC sup: monocytes cultured in DC differentiating medium in presence of supernatant collected from UC-MSC cultured alone.

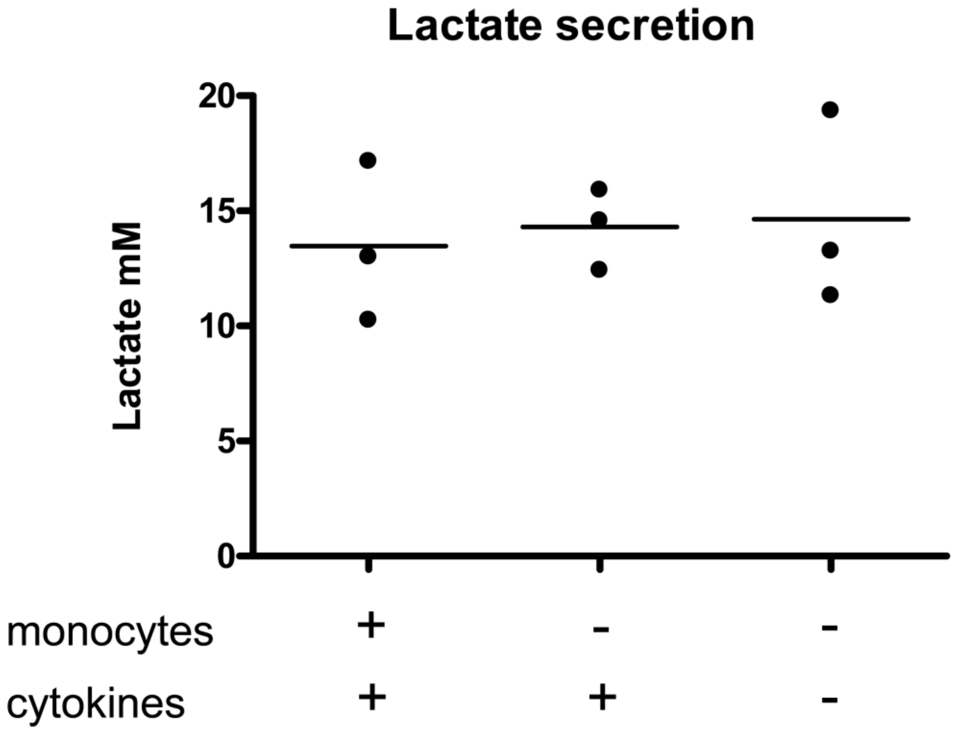

Supplementary Figure 9: UC-MSC were cultured in presence or absence of monocytes and the cytokines provided for DC differentiation (GM-CSF and IL-4). Half of the cell culture medium was replaced with fresh medium every 2-3 days, as in the protocol followed for DC differentiation. Lactate secretion was quantified at day 7 of culture. There is no significant difference between the amounts of lactate secreted in the different culture conditions.

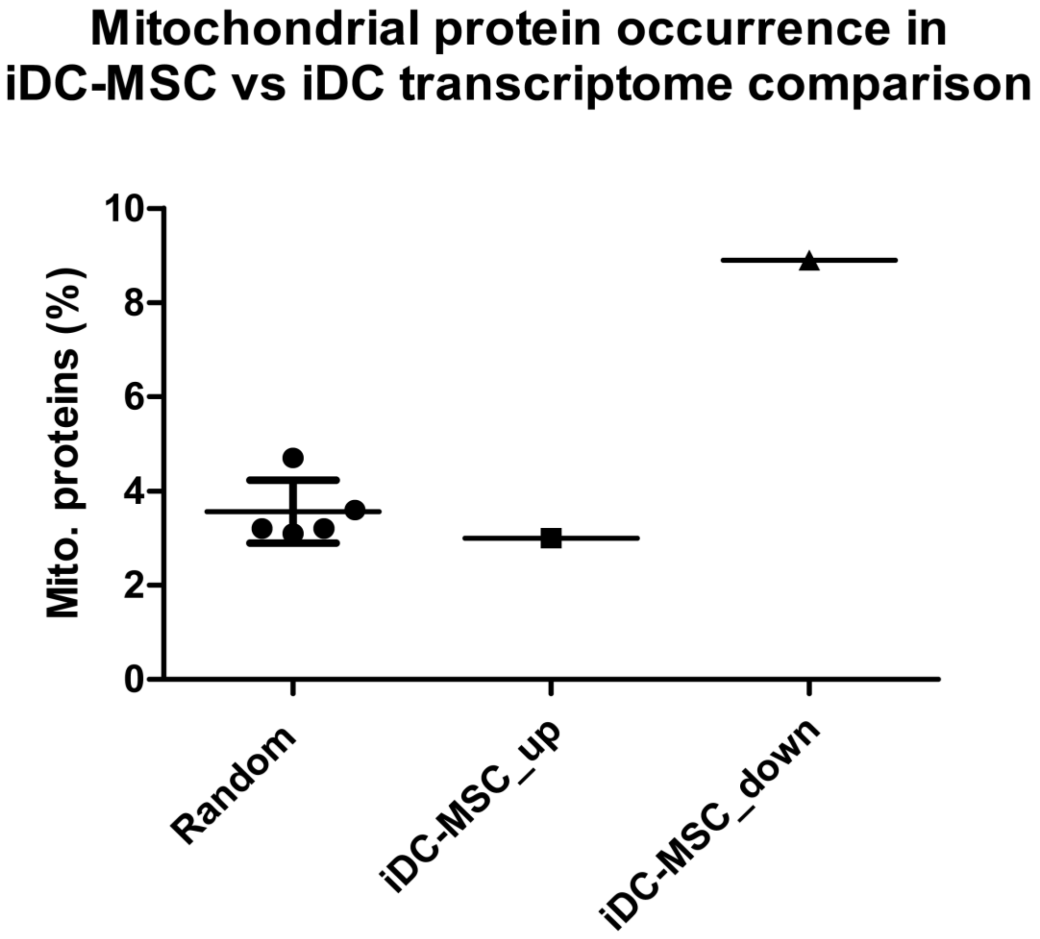

Supplementary Figure 10: Enrichment analysis for mitochondrion-expressed gene products in differential transcripts of the iDC versus iDC^{MSC} transcriptome comparison. Lists of non-redundant differentially-expressed genes (FDR≤0.01, Array signal>60) from the iDC versus iDC^{MSC} transcriptome comparison were queried for enrichment of mitochondrion-expressed gene products as provided in the MitoProteome database [42]. Lists of randomly-chosen genes (n=5, 1000 genes per list) were also queried using the MitoProteome database to establish the background percentage (%) of occurrence and the standard deviation (bars). The list of gene

transcripts that were downregulated in iDC^{MSC} (down) compared to iDC contained a higher percentage of mitochondrial genes than by chance.
